# Three spider species of the genus *Mimetus* Hentz, 1832 (Araneae, Mimetidae) from China

**DOI:** 10.3897/zookeys.626.7918

**Published:** 2016-10-25

**Authors:** Chen Zeng, Cheng Wang, Xian-Jin Peng

**Affiliations:** 1 College of Life Sciences, Hunan Normal University, Changsha, Hunan 410081, China; 2 College of Biological, Agricultural and Forest Engineering, Tongren, Guizhou 554300, China

**Keywords:** Taxonomy, new species, Asia, diagnosis, redescription

## Abstract

The present paper deals with three species of the genus *Mimetus* from China, including *Mimetus echinatus* Wang, 1990, *Mimetus lamelliformis* **sp. n.** (male), and *Mimetus wangi* **sp. n.** (female and male). *Mimetus lamelliformis* differs from the related species *Mimetus echinatus* Wang, 1990 by: cymbial tip with several slender long macrosetae; cymbium boat-shaped, length/width ratio about 3/1 in retrolateral view; vexillum about 1/2 length of cymbium in retrolateral view. *Mimetus wangi* **sp. n.** differs from the related species *Mimetus sinicus* Song & Zhu, 1993 by: the opisthosoma with a pair of distinct outgrowths in the dorsum; sperm duct nearly horizontal; spermathecae kidney shaped and contiguous. Photos of body and copulatory organs, line drawings of copulatory organs, as well as the locality map are provided.

## Introduction

The genus *Mimetus* was established by Hentz (1832) with the type species *Mimetus syllepsicus* Hentz, 1832. A total of 54 species have been described from all over the world except Australia and Antarctica. Up to now, only six species have been recorded from China by Wang (1990), Liang and Wang (1991), Song and Zhu (1993) (World Spider Catalog 2016). *Mimetus* species can be distinguished from members of other genera by the male bearing a “shovel” (a shovel-like appendage on the dorsal edge of the cymbium), and a “vexillum” (the distal sclerotized extension of the shovel) (Heimer 1986), in combination with a bulb possessing sclerites S2–S5 and three longitudinal lines of spines on the carapace (Harms and Dunlop 2009). Female epigyne simple but distinct, with two inconspicuous copulatory opening, spermatheca strongly sclerotized (Harms and Harvey 2009a, b).

While examining specimens collected from Hunan, Guizhou and Yunnan Provinces, two members of *Mimetus* were identified as new species and one was identified to be *Mimetus echinatus* Wang, 1990. Descriptions and diagnoses of the new species and a redescription of *Mimetus echinatus* have been presented in this paper.

## Material and methods

All specimens were kept in 75% ethanol, examined and measured with an Olympus SZX16 stereomicroscope and an Olympus BX53 compound microscope, respectively. Photos were taken with a digital camera Canon Powershot G12 mounted on an Olympus SZX16 and compound focus images were generated using Helicon Focus Software (3.10).

Specimens are deposited in the College of Life Sciences, Hunan Normal University, Changsha, China. All measurements are given in millimeters (mm). Leg measurements are given as: total length (femur, patella + tibia, metatarsus, tarsus). The abbreviations used in text including:

AER
 anterior eye row 


ALE
 anterior lateral eye 


AME
 anterior median eye 


CD
 copulatory duct 


CO
 copulatory opening 


E
 embolus 


FD
 fertilization duct 


M
 membrane 


MOA
 median ocular area 


P
 paracymbium 


PER
 posterior eye row 


PLE
 posterior lateral eye 


PME
 posterior median eye 


S
 spermatheca 


SH
 shovel 


ST
 subtegulum 


VE
 vexillum 


## Taxonomy

### *Mimetus* Hentz, 1832

#### Mimetus echinatus

Taxon classificationAnimaliaAraneaeMimetidae

Wang, 1990

Figs 1 , 2 , 3 , 4

Mimetus echinatus Wang, 1990: 44, fig. IV.6–10 (male and female).

##### Type material examined.

2♂, 1♀, **China, Hunan**, Changsha City, Yuelu Mountain, 20 April 1981, Jiafu Wang leg.;

##### Other material examined.

6♂, 2♀, **China, Hunan**, Shimen County, Huping Township, Daling Village, 30.02175°N, 110.37455°E, 710m, 19 June 2014, Cheng Wang, Bing Zhou, Jiahui Gan and Yuhui Gong leg.

##### Redescription.

**Figure 1.**
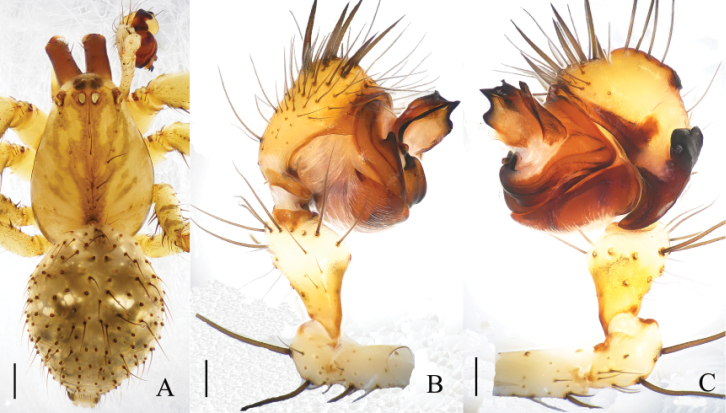
*Mimetus echinatus* Wang, 1990, one of the males. **A** habitus, dorsal view **B** palp, prolateral view **C** palp, retrolateral view. Scale bars: **A**, 1.0; **B–C**, 0.2.

**Figure 2.**
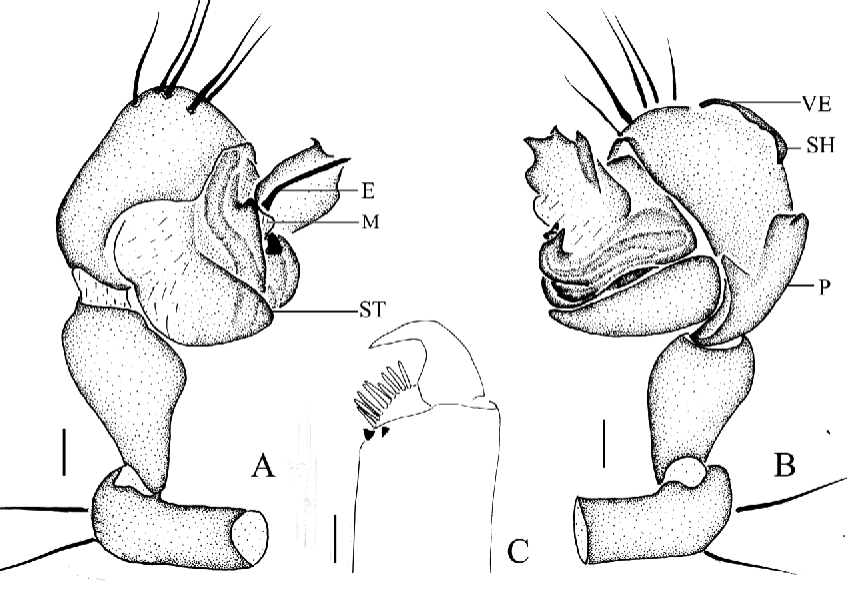
*Mimetus echinatus* Wang, 1990, one of the male. **A** palp, prolateral view **B** palp, retrolateral view **C** left chelicera, ventral view. Scale bars: **A–B**, 0.2; **C**, 0.1.

**Male.** Carapace (Fig. 1A) bright yellow, long oval, widest at coxae II and III. Fovea circular, deep, its surrounding area reddish brown. Sternum light yellow, pear-shaped, the surrounding with seven gray circular patches, margin with long scopulae, median glabrous. AER slightly recurved, PER nearly straight, ALE and PLE contiguous. Chelicerae reddish brown, with 9 promarginal peg setae and 2 retromarginal teeth (Fig. 2C). Color of endites and labium similar. Legs spiniferous, femora with reddish brown patches. Dorsum of opisthosoma (Fig. 1A) suboval, light yellow-brown, with white patches on both sides, covered with long macrosetae. Venter grayish-white, with round white patches.

Male palp (Figs 1B–C, 2A–B): Patella strong, the dorsum with two thick and long macrosetae. Cymbium wedge, length/width ratio about 2/1, tip with several robust macro-setae Paracymbium distinct, distal black and strongly sclerotized. Distal division of the bulb rolls up as sulciform to protect embolus and serves as a functional conducter. Embolus relatively slender, distal division reaches the position of 2:00 o’clock approximately with a membrane covering its proximal area in prolateral view.

**Figure 3.**
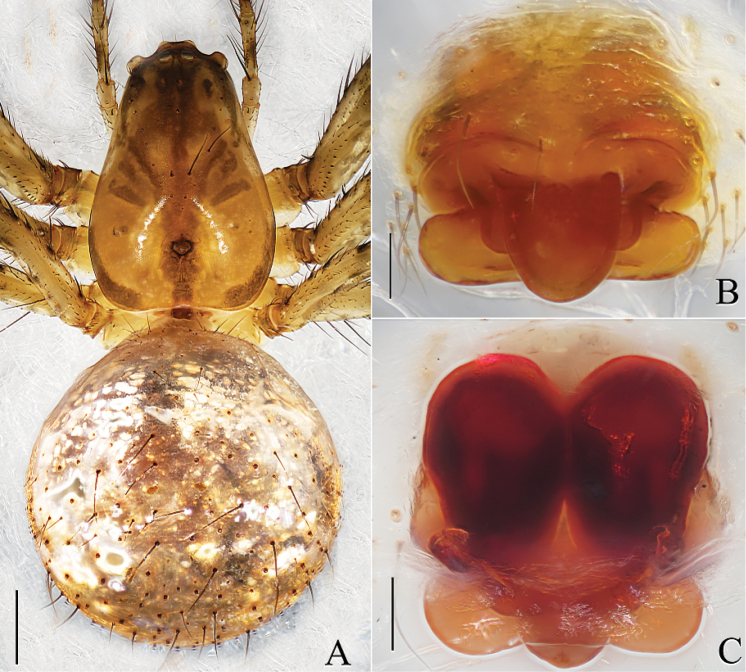
*Mimetus echinatus* Wang, 1900, from Shimen. **A** habitus, dorsal view **B** epigyne, ventral view **C** vulva, dorsal view. Scale bars: **A**, 1.0; **B–C**, 0.1.

**Figure 4.**
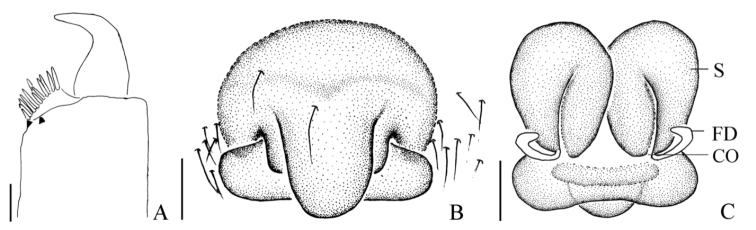
*Mimetus echinatus* Wang, 1990, from Shimen. **A** left chelicerae, ventral view **B** epigynum, ventral view **C** vulva, dorsal view. Scale bars: **A–C**, 0.1.

**Female.** Carapace (Fig. 3A) yellowish brown, pear-shaped, with several reddish brown diagonal patches, posterior margin reddish brown, fovea and sternum similar to male. ALE and PLE contiguous, AER and PER nearly straight. Endite, labium and sternum similar to male but darker. Chelicerae stronger than male with 12 promarginal peg setae and 2 retromarginal teeth (Fig. 4A). Legs femur with reddish annuli basely, other area with small reddish brown patches, patella brown, metatarsus with brown annuli. Opisthosoma (Fig. 3A) subcircular, slightly wider than long. Dorsum of opisthosoma (Fig. 3A) yellowish brown, with long macrosetae and white patches. The markings of venter similar to male. Spinnerets reddish brown, anterior spinnerets longest.

Epigyne (Figs 3B–C, 4B–C) slightly wider than long, with three parts, including lingulate scape, rectangular basal plate and a laminar layer covering the venter of the basal plate. Scape and basal plate cross shaped, scape about 1/2 length of Epigyne, width of basal plate approximately equal to Epigyne. Spermathecae ovoid, about 2/3 length of epigynum. Copulatory ducts indistinct in dorsal view.

##### Distribution.

China (Hunan).

#### Mimetus lamellaris sp. n.

Taxon classificationAnimaliaAraneaeMimetidae

http://zoobank.org/F3A39691-1AAA-41EA-B389-9538A19E0274

Figs 5 , 6

##### Type material.

**Holotype** ♂, **China, Guizhou**: Yanhe County, Daheba Township, Mayanhe National Nature Reserve 28.65839°N, 108.26033°E, 364m, 28 July 2014, Xianjin Peng, Cheng Wang, Bing Zhou, Ping Liu, Yi Huang and Mingyong Liao leg.

##### Etymology.

The specific name comes from the Latin word *lamellaris*, meaning flaky and referring to the flaky vexillum on the cymbial tip; adjective.

##### Diagnosis.

**Figure 5.**
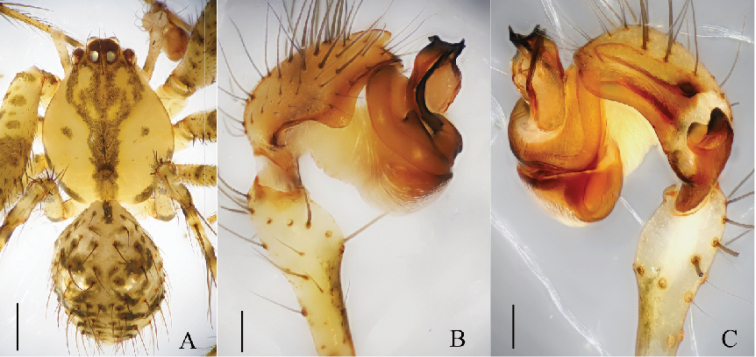
*Mimetus lamellaris* sp. n., holotype male. **A** habitus, dorsal view **B** palp, prolateral view **C** palp, retrolateral view. Scale bars: **A**, 0.5; **B–C**, 0.1.

**Figure 6.**
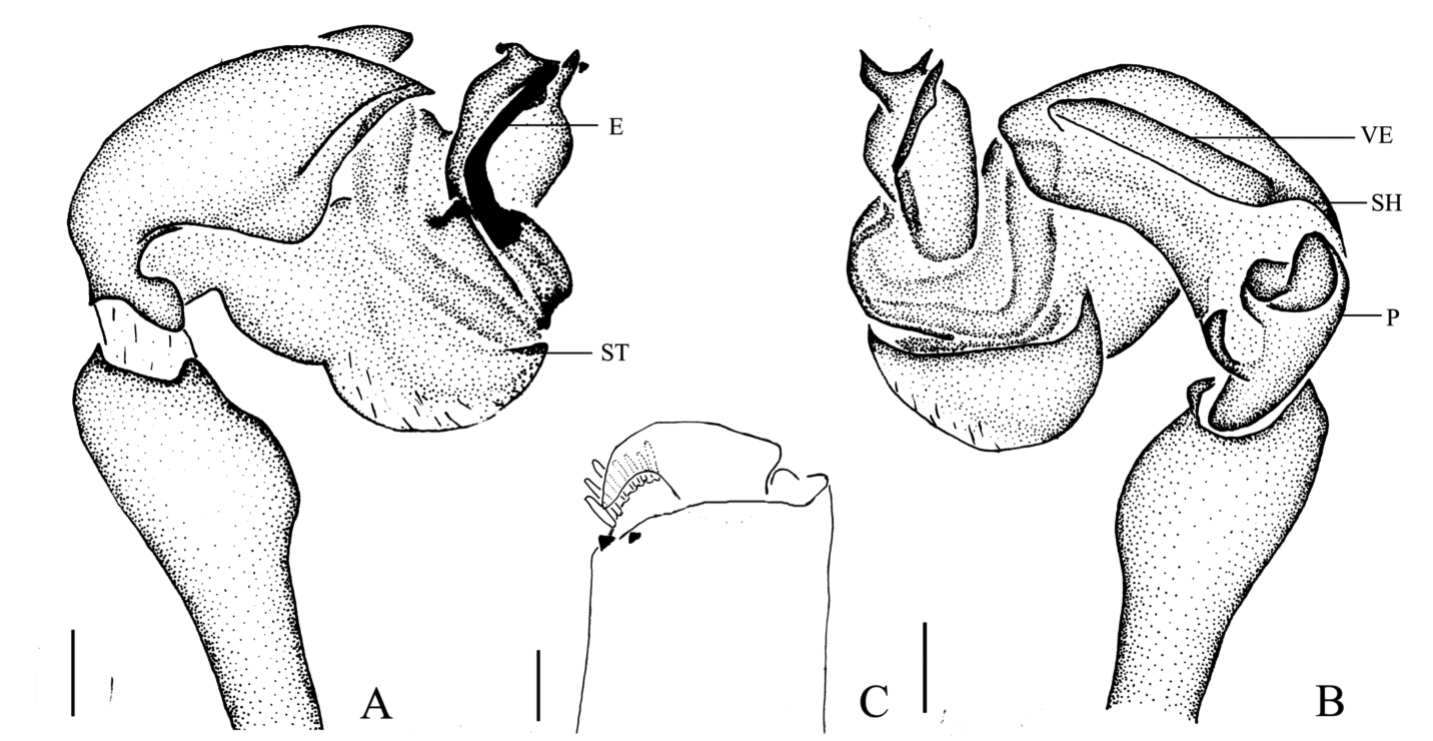
*Mimetus lamellaris* sp. n., holotype male. **A** palp, prolateral view **B** palp, retrolateral view **C** left chelicerae, ventral view. Scale bars: **A–C**, 0.1.

The new species can be distinguished from all known congeneric species by: cymbial tip with several slender long macrosetae (Fig. 5B–C); cymbium boat-shaped, length/width ratio about 3/1 in retrolateral view (Figs 5C, 6B); the angle between the basal and apical of embolus about 110° (Figs 5B, 6A); vexillum about 1/2 length of cymbium in retrolateral view (Figs 5B–C, 6B–C); apical part of paracymbium broaden to rotund, with two hornlike outgrowths (Figs 5C, 6B).

##### Description.

**Male**: Total length 3.05. Prosoma 1.65 long, 1.30 wide. Opisthosoma 1.40 long, 1.13 wide. Clypeus 0.20 high. Carapace (Fig. 5A) yellow, long oval, widest at coxae II and III, with a longitudinal brown patch medially, posterior partion reddish brown. Fovea circular, deep, surrounding area reddish brown. AER recurved, PER nearly straight, ALE and PLE continuous. Eye sizes and interdistances: AME 0.13, ALE 0.08, PME 0.11, PLE 0.05, AME–AME 0.06, ALE–AME 0.07, PME–PME 0.06, PME–PLE 0.13. MOA anterior width 0.34, posterior 0.28, length 0.35. Chelicerae reddish brown, with 7 promarginal peg setae and 2 retromarginal teeth (Fig. 6C), distal area with long hairs. Endites light yellow, longer than wide. Labium reddish brown, wider than long. Sternum yellow, long oval, each side with two brown patches, margins with long scopulae, centrally glabrous. Legs light yellow, spiniferous, with reddish brown patches equidistributed. Length of legs: I 10.35 (2.73, 3.52, 2.60, 1.50), II 7.78 (2.43, 2.72, 12.6, 1.01), III 4.53 (1.10, 1.53, 1.00, 0.90), IV 5.66 (1.12, 2.03, 1.11, 0.60). Leg formula: 1243. Dorsum of opisthosoma (Fig. 5A) light yellow, long oval, with long macrosetae and brown patches. Venter gray white, with white patches in middle.

Male palp (Figs 5B–C, 6A–B): tibia long and thin, distally swollen, with several long macrosetae. Cymbium boat-shaped, widest in middle in prolateral view, with slender macrosetae on the tip. Vexillum flaky, about 1/2 length of cymbium in retrolateral view, visible in prolateral view. Distal of paracymbium broaden to rotund, with two hornlike outgrowths. Basal division of the bulb visible, distal division of the bulb rolls up as sulciform to protect embolus. Embolus hook-shaped, and the angle between its basal and apical about 110°.

**Female.** Unknown.

##### Distribution.

China (Guizhou).

#### Mimetus wangi sp. n.

Taxon classificationAnimaliaAraneaeMimetidae

http://zoobank.org/35717222-6AAA-402E-800C-1E4C46CFE6CF

Figs 7 , 8 , 9 , 10

##### Type material.

**Holotype** ♂, **China, Yunnan**: Gaoligong Mountains, Dulongjiang Township, Xianjiudang Village, 27.93682°N, 98.3260°E, 1634m, 5 April 2004, Guo Tang leg. **Paratypes**: 5♀, same data as holotype.

##### Etymology.

The specific name is a patronym in honor of Professor Jiafu Wang, a well known spider taxonomist in China; noun.

##### Diagnosis.

**Figure 7.**
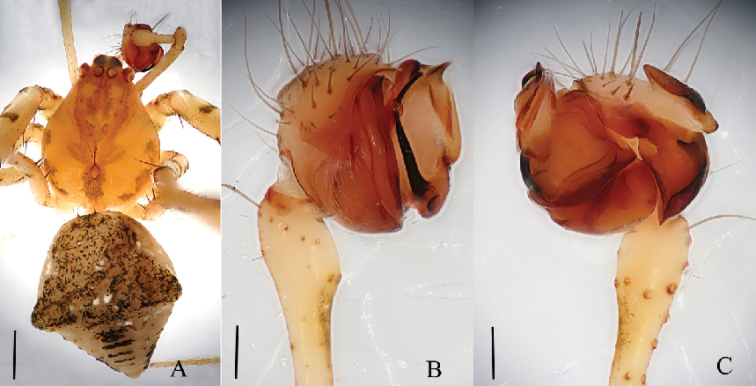
*Mimetus wangi* sp. n., holotype male. **A** habitus, dorsal view **B** palp, prolateral view **C** palp, retrolateral view. Scale bars: **A**, 0.5; **B–C**, 0.1.

**Figure 8.**
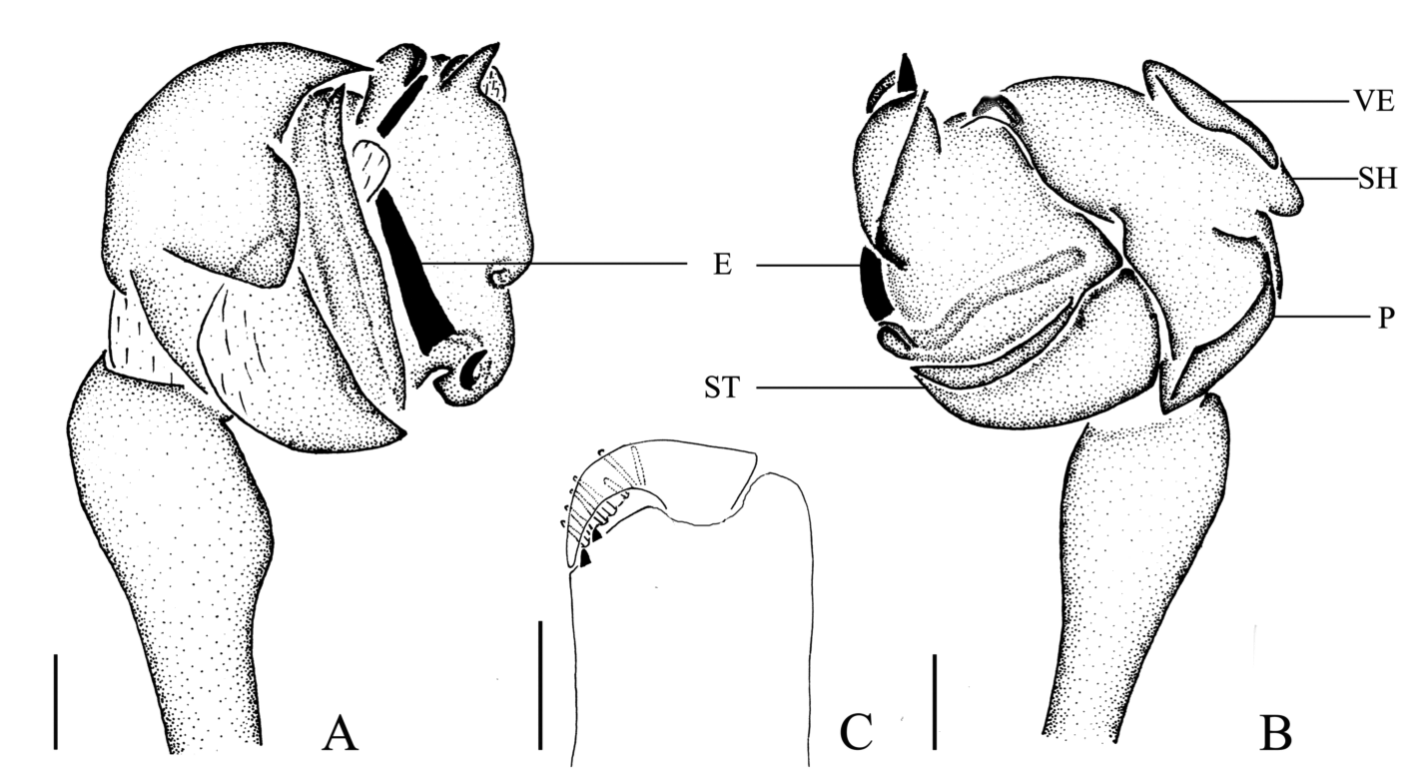
*Mimetus wangi* sp. n., holotype male. **A** palp, ventral view **B** palp, prolateral view **C** left chelicera, ventral view. Scale bars: **A–B**, 0.1; **C**, 0.1.

**Figure 9.**
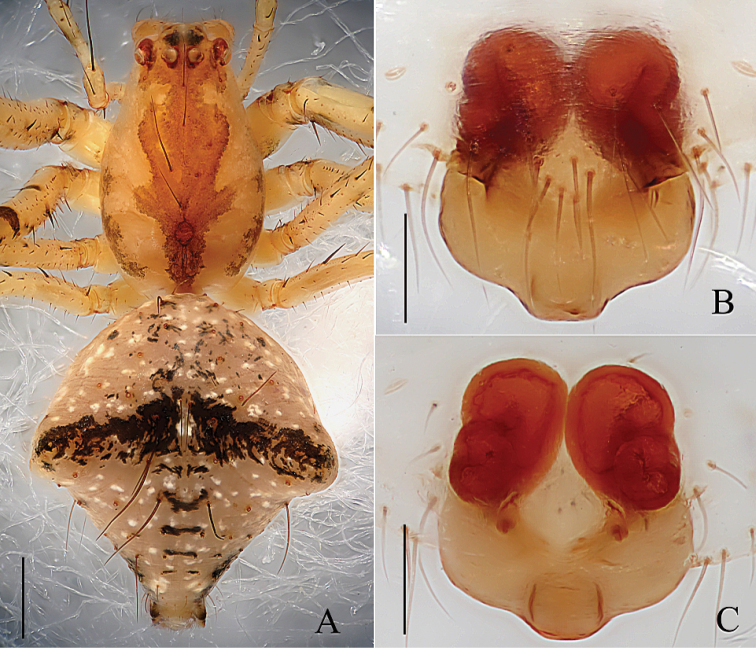
*Mimetus wangi* sp. n., one of the female paratypes. **A** habitus, dorsal view **B** epigyne, ventral view **C** vulva, dorsal view. Scale bars: **A**, 0.5; **B–C**, 0.1.

**Figure 10.**
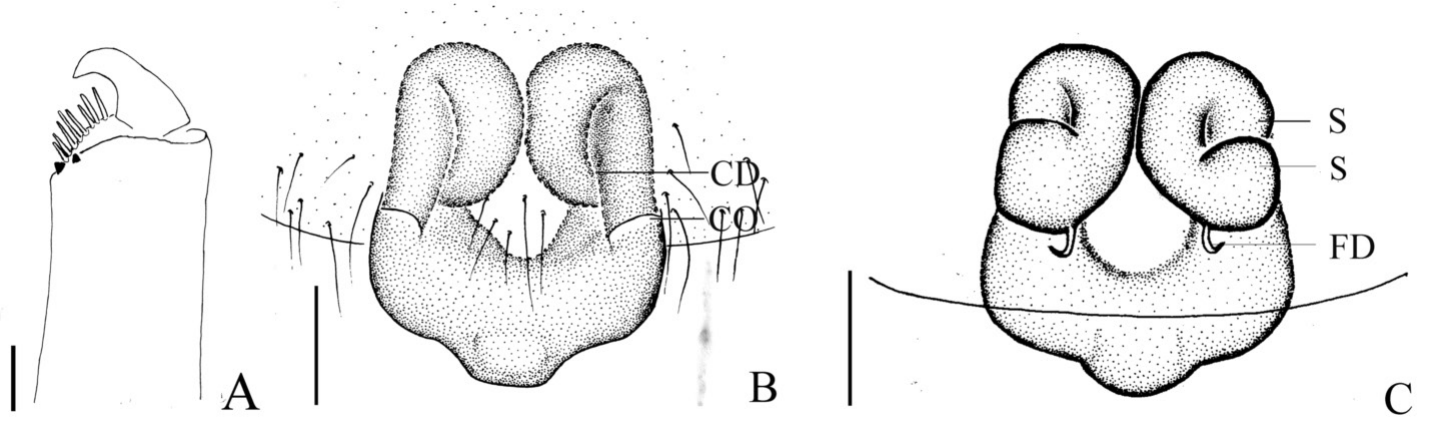
*Mimetus wangi* sp. n., one of the female paratypes. **A** left chelicerae, ventral view **B** epigynum, ventral view **C** vulva, dorsal view. Scale bars: **A–C**, 0.1.

The new species can be distinguished from all known congeneric species by: the dorsum of the opisthosoma with a pair of distinct outgrowths (Fig. 7A); the ratio of cymbium length/width about 2/1 in retrolateral view (Figs 7C, 8B); spermathecae kidney shaped (Figs 9C, 10C) and contiguous (Figs 9B, 10B); the width of spermathecae slightly narrower than basal plate (Figs 9B, 10B).

##### Description.

**Male**: Total length 3.34. Prosoma 1.50 long, 1.20 wide. Opisthosoma 1.84 long, 1.40 wide. Clypeus 0.05 height. Carapace (Fig. 7A) yellow brown, long oval, widest at coxae II and III, with longitudinal brown patches at median area and three brown patches on both sides of the lateral margins. Fovea circular. AER slightly recurved, PER nearly straight, ALE and PLE contiguous. Eye sizes and interdistances: AME 0.13, ALE 0.08, PME 0.10, PLE 0.06, AME–AME 0.08, ALE–AME 0.05, PME–PME 0.05, PME–PLE 0.13, MOA anterior width 0.28, posterior width 0.25, length 0.31. Chelicerae yellow, with 7 promarginal peg setae and 2 retromarginal teeth. Endites light yellow, longer than wide. Labium light yellow, longer than wide. Sternum pear-shaped, glabrous, colored as labium except margin with few macrosetae. Legs slim, spiniferous, with brown patches. Length of legs: I 11.23 (3.31, 3.82, 2.80, 1.30), II 9.00(2.48, 2.91, 2.40, 1.21), III 4.58 (1.01, 1.68, 1.00, 0.0.88), IV 6.05 (1.90, 2.00, 1.40, 0.75). Leg formula: 1243. Opisthosoma (Fig. 7A) long oval, filled with small black spots and few white patches, anterior area with few macrosetae, middle portion widest, with two outgrowths in both sides, posterior portion sloping, with five transverse black stripes. Venter with three brown patches, median area grey, glabrous, with white spots on both sides.

Male palp (Figs 7C–D, 8B–C): tibia slim, with several macrosetae. Cymbial length/width ratio about 2/1 in retrolateral view, distal end extending to vexillum, shovel obvious, with dense long setae. Sperm duct nearly horizontal. Embolus with a membrane covering its terminal 2/3 portion in prolateral view. Paracymbium massive.

**Female.** Total length 3.65. Prosoma 1.55 long, 1.10 wide. Opisthosoma 1.95 long, 2.05 wide. Clypeus 0.10 high. Carapace (Fig. 9A) yellowish brown, long oval. AER recurved, PER slightly procurved. ALE and PLE continuous. Eye sizes and interdistances: AER 0.10, ALE 0.13, PME 0.13, PLE 0.15, AME–AME 0.04, AME–ALE 0.06, PME–PME 0.05, PME–PLE 0.10. MOA anterior width 0.28, posterior width 0.21, length 0.31. Chelicerae, endites and labium coloured as in male, labium slightly wider than long. Chelicerae with 8 promarginal peg setae and 2 retromarginal teeth. Sternum similar to male except for lightly colored. Leg patches and spines similar to male.

Length of legs: I 12.92 (3.51, 4.30, 3.60, 1.50), II 9.55 (2.87, 2.91, 2.38, 1.38), III 5.93 (1.82, 1.91, 1.21, 1.00), IV 6.75 (2.10, 2.30, 1.35, 1.00). Dorsum of opisthosoma (Fig. 9A) similar to male, except for fewer black spots and more white spots, and between the two outgrowths is a transverse black wide stripe. Venter similar to male except three patches darker.

Epigyne (Figs 9B–C, 10B–C) slightly longer than wide, with a yellowish brown labiate outgrowth at the bottom of the base plate, copulatory openings visible, close to epigastric furrow. Spermathecae kidney shaped, contiguous, slightly narrower than basal plate. Basal plate scalloped. Copulatory ducts indistinct in dorsal view.

**Figure 11.**
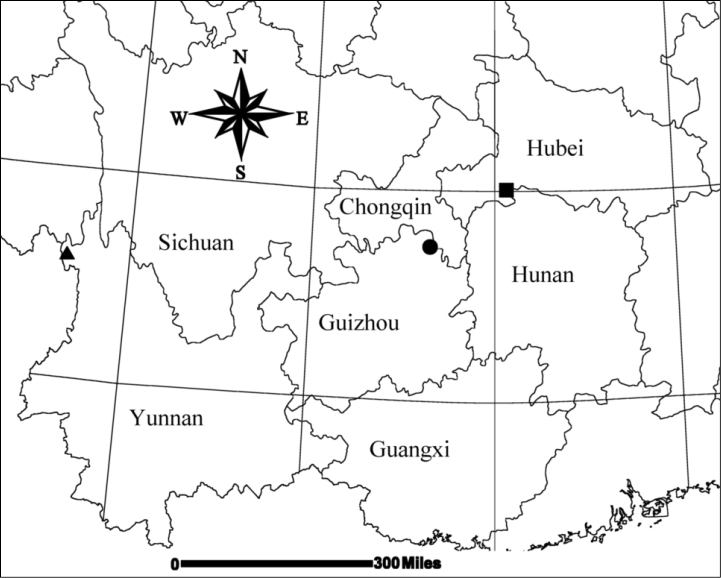
Collecting localities of three *Mimetus* species. ■ *Mimetus echinatus*; ● *Mimetus lamellaris* sp. n.; ▲ *Mimetus wangi* sp. n..

##### Distribution.

China (Yunnan).

## Supplementary Material

XML Treatment for Mimetus echinatus

XML Treatment for Mimetus lamellaris

XML Treatment for Mimetus wangi
